# Validation of the Investigator 24plex QS Kit: a 6-dye multiplex PCR assay for forensic application in the Chinese Han population

**DOI:** 10.1080/20961790.2019.1665160

**Published:** 2019-12-06

**Authors:** Ruiyang Tao, Chong Chen, Xiang Sheng, Ruocheng Xia, Xiaochun Zhang, Jingyi Zhang, Zihao Yang, Suhua Zhang, Chengtao Li

**Affiliations:** a Institute of Forensic Medicine, West China School of Basic Medical Sciences & Forensic Medicine, Sichuan University, Chengdu, China; b Shanghai Key Laboratory of Forensic Medicine, Shanghai Forensic Service Platform, Academy of Forensic Sciences, Ministry of Justice, Shanghai, China; c College of Medicine and Forensics, Xi’an Jiaotong University Health Science Center, Xi’an, China; d QIAGEN, Hilden, Germany; e Department of Forensic Medicine, School of Basic Medical Science, Wenzhou Medical University, Wenzhou, China; f Department of Forensic Science, Medical School of Soochow University, Suzhou, China

**Keywords:** Forensic sciences, forensic genetics, short tandem repeat (STR) typing, Investigator 24plex QS Kit, validation study

## Abstract

The Investigator 24plex QS Kit (QIAGEN, Hilden, Germany) is a 6-dye fluorescent chemistry short tandem repeat (STR) polymerase chain reaction (PCR) amplification system that simultaneously amplifies 20 of the expanded Combined DNA Index System (CODIS) core STR loci, SE33, DYS391, and the standard sex-determining locus, amelogenin, as well as two special internal performance quality sensor controls (QS1 and QS2), which are included in the primer mix to check the PCR performance. This study was designed to be a pilot evaluation of this STR-PCR kit in a Chinese Han population regarding the PCR conditions, sensitivity, precision, accuracy, repeatability, reproducibility, and concordance; tolerance to PCR inhibitors; applicability to real “forensic-type” samples; species specificity; mixture, balance and stutter analyses, and utility in a population investigation. The exhaustive validation studies demonstrated that the Investigator 24plex QS system is accurate, sensitive and robust for STR genotyping. In addition, these genetic markers in the population data in our study indicated that they can also be useful for forensic identification and paternity testing in the Chinese Han population.

## Introduction

Since the 1990s, short tandem repeat (STR) analysis, based on capillary electrophoresis (CE) technology, had been recognized as a standard approach for human identification and paternity testing worldwide [1–3]. Multiplex amplification of widely used STR markers makes it convenient to establish centralized STR databases in different counties throughout the world, while the valuable genetic information provided by databases also makes STR technology powerful, as it can help to directly search and match STR profiles generated from crime scenes. To increase the power of discrimination for individual identification and to facilitate international compatibility, the Federal Bureau of Investigation (FBI) expanded the Combined DNA Index System (CODIS) core STR loci from the original 13 loci to 20 loci: D1S1656, D2S441, D2S1338, D3S1358, D5S818, D7S820, D8S1179, D10S1248, D12S391, D13S317, D16S539, D18S51, D19S433, D21S11, D22S1045, CSF1PO, FGA, TH01, TPOX and vWA [4,5].

Using the current 6-dye labeling technology, the Investigator 24plex QS Kit (QIAGEN, Hilden, Germany) was developed by combining 20 expanded CODIS loci, one Y-STR (DYS391), the SE33 locus (highly recommended), amelogenin, and two internal performance controls, Quality Sensors (QS1 and QS2) [6]. These internal controls include a 70-bp and 435-bp PCR fragment, which are included in the Primer Mix and simultaneously amplified with input DNA to monitor PCR performance, distinguish failed PCR progress resulting from a lack of qualitied DNA, and differentiate sample DNA from degraded DNA [7]. According to Kraemer et al. [8], this system was developed for detecting challenging, low quality and low quantity samples for forensic casework. Although validation studies on this STR typing system have been conducted in several forensic laboratories, these studies had not been conducted in the Chinese population.

To understand the availability and practicality of the Investigator 24plex QS Kit in Chinese populations, we evaluated the overall performance of the kit by following the *Validation Guidelines for DNA Analysis Methods* (2016) issued by the Scientific Working Group on DNA Analysis Methods (SWGDAM) [9] and the Chinese National Standard (CNS) *Basic Quality Requirements of Forensic Science Human Fluorescent STR Multiplex PCR Testing Reagent* (GA/T815-2009) [10]. As such, the PCR conditions, sensitivity, precision/accuracy, repeatability/reproducibility, concordance, stability, performance of genuine “forensic-type” samples, species specificity, mixture analysis, balance, stutter analysis, as well as population genetics were investigated in this study. The results illustrated that the Investigator 24plex QS Kit is sensitive and reliable in forensic application and, particularly, that the included Quality Sensors can provide useful information for troubleshooting strategies. Furthermore, these STR markers are informative in the Chinese Han population and have a robust individual identification capability, non-parent exclusion probability, and database comparison compatibility.

## Materials and methods

### Sample preparation and DNA extraction

Peripheral blood samples from 500 healthy, unrelated Chinese Han individuals (250 females and 250 males) were collected after receiving written informed consent. DNA isolation was carried out by using the QIAamp DNA Blood Mini Kit (QIAGEN), and the quantity of genomic DNA was detected using a NanoDrop 2000 spectrophotometer (NanoDrop Technologies, Inc., Wilmington, DE, USA) in accordance with the manufacturer’s protocol. DNA from the 9947A, 9948, 007 (Thermo Fisher Scientific, Waltham, MA, USA) and 2800M (Promega, Madison, WI, USA) human cell lines were used as positive controls. DNA samples were diluted to 0.5 ng/µL, or the appropriate concentration, using Tris-EDTA (TE) buffer.

### PCR amplification and capillary electrophoresis (CE)

Amplification was performed in a single multiplex PCR system using 7.5 µL Fast Reaction Mix 2.0, 2.5 µL Primer Mix, 0.5 ng genomic DNA and appropriate amount of water for a final reaction volume of 25 µL. PCR was performed on the GeneAmp 9700 PCR system (Thermo Fisher) with a Gold-plated Silver 96 Well Block in the “Max Mode”, including 3 cycles at 98 °C for 30 s, 64 °C for 55 s and 72 °C for 5 s; 27 cycles at 96 °C for 10 s, 61 °C for 55 s and 72 °C for 5 s; a final extension at 68 °C for 2 min and then held at 60 °C for 2 min.

Amplified products were analyzed by adding 1 µL of each PCR product to 12 µL of a 1:24 mixture of BTO_550 size standard (60, 80, 90, 100, 120, 140, 160, 180, 200, 220, 240, 250, 260, 280, 300, 320, 340, 360, 380, 400, 425, 450, 475, 500, 525, and 550 bp) (QIAGEN) and Hi-Di formamide (Thermo Fisher) for CE detection. The mixture was then denatured by heating to 95 °C for 3 min and cooling to 4 °C for 3 min. Samples were injected at 1.6 kV for 33 s and electrophoresed at 13 kV for 1 550 s using a run temperature of 60 °C, the BT6 filter set and POP4 polymer (Thermo Fisher) on the 3500xL Genetic Analyzer (Thermo Fisher). Genotyping data were collected and analyzed by the GeneMapper^®^ *ID-X* Software v1.5 (Thermo Fisher). Peak detection default threshold of the analysis software, above 100 Relative Fluorescence Units (RFUs), was applied for analysis.

### Validation studies

#### PCR condition tests

To evaluate the parameter range of PCR conditions that can produce reliable STR genotyping results, different annealing temperatures (57 °C, 59 °C, 61 °C, 63 °C, and 65 °C) and varying numbers of PCR cycles (25, 27 and 29) were performed in triplicate with 0.5 ng positive control DNA from the 9948 human cell line. These varying conditions were tested in a series of PCR reactions in which only one parameter was altered and others remain as suggested in the protocol.

#### Accuracy study and sizing precision

For the accuracy study, 200 random individual DNA samples were genotyped under the standard conditions on the 3500xL Genetic Analyzer. Based on the genotyping information, each allele size was compared to that of the corresponding allelic ladder to calculate the size difference.

For the precision study, the fragment size was measured *via* CE for the 24 allelic ladder samples on the 3500xL Genetic Analyzer. Then, the average fragment length and standard deviation of each allele were calculated.

#### Repeatability, reproducibility and concordance tests

The genotyping profiles of 50 individual DNA samples were compared to their replicate results to evaluate the repeatability of the kit. These samples were also amplified and genotyped in triplicate in an independent accredited lab to test the reproducibility. Since the amelogenin and 22 STR loci in the Investigator 24plex QS Kit are also contained in the GlobalFiler™ Kit (Thermo Fisher), the aforementioned 50 DNA samples were subsequently genotyped using the latter kit for the concordance test of the 24plex system.

#### “Forensic-type” samples

In forensic casework, it is often necessary to handle various of biological tissues, such as hair, nail, bone, and blood. Therefore, a series of different sample types, including saliva, saliva stain, buccal swab, blood stain on an FTA card, 10-year-old blood stain on gauze, FFPEB (formalin-fixed and paraffin-embedded biopsy) sample, hair roots, nail, vaginal secretion, menstrual blood, semen, semen stain, muscle, and bone were chosen and amplified in triplicate to test the ability of the Investigator 24plex QS Kit to process various sample types.

#### Sensitivity

To assess the optimal quantity and variable range of DNA input for the 24plex panel, a serial dilution of the positive control DNA 9948 was amplified in triplicate with quantities of 5 ng, 2 ng, 1 ng, 500 pg, 250 pg, 125 pg, 62.5 pg, 31.25 pg and 15.625 pg DNA. The average percentage of the detected loci and the average peak heights in each of the above tests were measured. In this part, control DNA 9948 was quantified by Qubit^®^ dsDNA HS Assay Kit on a Qubit^®^ 2.0 Fluorometer (Thermo Fisher) according to the manufacturer’s protocol.

#### Stability

The stability study was performed using different concentrations of the typical PCR-inhibitors hematin, humic acid, indigotin, nigrosine and urea (Aladdin Co Ltd, Hangzhou, China) that were co-amplified with positive control DNA 9948 via the 24plex system. The inhibitor concentrations ranged as follows: 100 µmol/L, 300 µmol/L, 500 µmol/L, 700 µmol/L, and 900 µmol/L of hematin; 20 ng/µL, 40 ng/µL, 80 ng/µL, 160 ng/µL, and 320 ng/µL of humic acid; 2 000 ng/µL, 4 000 ng/µL, 8 000 ng/µL and 16 000 ng/µL of indigotin; 40 ng/µL, 60 ng/µL, 80 ng/µL, 100 ng/µL, 120 ng/µL and 150 ng/µL of nigrosine; and 2 000 ng/µL, 4 000 ng/µL, 8 000 ng/µL, 16 000 ng/µL, 32 000 ng/µL and 48 000 ng/µL of urea. Each reaction was amplified in triplicate.

#### Species specificity

For the species specificity test, 2 ng DNA samples from 11 species of mammals (monkey, cow, horse, donkey, deer, sheep, pig, rabbit, dog, cat, and rat), four species of non-mammals (fish, snake, chicken and duck) and three microbial species (*Escherichia coli*, *Staphylococcus albus* and *Staphylococcus aureus*) were subjected to PCR amplification using the Investigator 24plex QS Kit in triplicate.

#### Mixture study

At serial ratios (1:1, 1:3, 3:1, 1:9, 9:1, 1:19, and 19:1), 14 mixed samples were prepared using control DNA from the 9947A human cell line mixed with DNA from the 9948 human cell line and using the DNA from the 9948 cell line mixed with DNA from the 2800M cell line. Using the 24plex system, 1 ng of each mixture was amplified and detected in triplicate.

#### Balance and stutter analysis

The allele height information of the 200 DNA samples used in *Section repeatability, reproducibility and concordance tests* was used to evaluate the balance performance and perform stutter analysis of the Investigator 24plex QS Kit. The balance of heterozygous alleles per locus was defined as the intra-locus balance; the balance of heterozygotes within one dye labelled loci mirrored the intra-colour balance; and the balance of heterozygotes within all analyzed loci among different STR profiles was regarded as inter-colour balance. Each calculation method has been described in a previous study [11]. For the stutter analysis, only peaks with one repeat smaller than the corresponding real allele were included in our study. The stutter ratio was calculated by dividing the peak height of the stutter peak by that of the allele.

### Population investigation and statistical analysis

The 500 unrelated Han individuals mentioned in *Section sample preparation and DNA extraction* were amplified and genotyped using the Investigator 24plex QS Kit. Hardy-Weinberg equilibrium (HWE) and linkage disequilibrium (LD) in the studied Han population were determined using Arlequin software v3.5.2 [12]. PowerStats V12.xls [13] was used to calculate the allele frequencies and other forensic parameters for these autosomal STR loci. According to the “Specification of parentage testing” (SF/ZJD0105001-2016), the total probability of discrimination power (TDP), the power of exclusion in duos (PE_D_) and trios (PE_T_), and the combined power of exclusion in duos (CPE_D_) and trios (CPE_T_) were counted. The allele frequencies of the DYS391 locus were calculated by direct counting. Gene diversity (GD), haplotype match probability (HMP) and discrimination capacity (DC) were calculated using the formulas GD = $N\times (1-\sum_{i=1}^{k}{p_{i}}^{2})/(N-1),$ HMP = $\sum_{i=1}^{k}{p_{i}}^{2},$ and DC =$\;k/\sum_{i=1}^{k}(p_{i}\times N),$ respectively, in which *p_i_* indicates the frequency of the *i*th haplotype, k indicates the number of haplotypes and *N* is the total number of individual samples.

## Results and discussion

### PCR condition tests

All alleles were detected at each of the different annealing temperatures (57 °C, 59 °C, 61 °C, 63 °C and 65 °C). The average peak height of the 23 loci ranged from 7 197.92 RFU (65 °C) to 16 413.73 RFU (61 °C); the latter was determined to be the ideal annealing temperature for genotyping (Supplementary Figure S1(A)). Complete profiles were obtained, and no allele dropout presented when the PCR cycle number ranged from 25 to 29. However, the average peak height for the 29 cycles reaction was too high (23 446.96 RFU) and produced spectral bleed-through. Therefore, the optimal cycle number of the 24plex system was considered to be 27 with a moderate average peak height of 16 282.96 RFU (Supplementary Figure S1(B)). These results were in accordance with those recommended in the user guide, and PCR was completed in less than 1 h, which aligns with forensic practice. The general characteristics of the 23 loci included in this system as well as the genotypes of the aforementioned four positive control DNA samples are shown in Supplementary Table S1.

### Sizing precision and accuracy study

**Figure 1.**
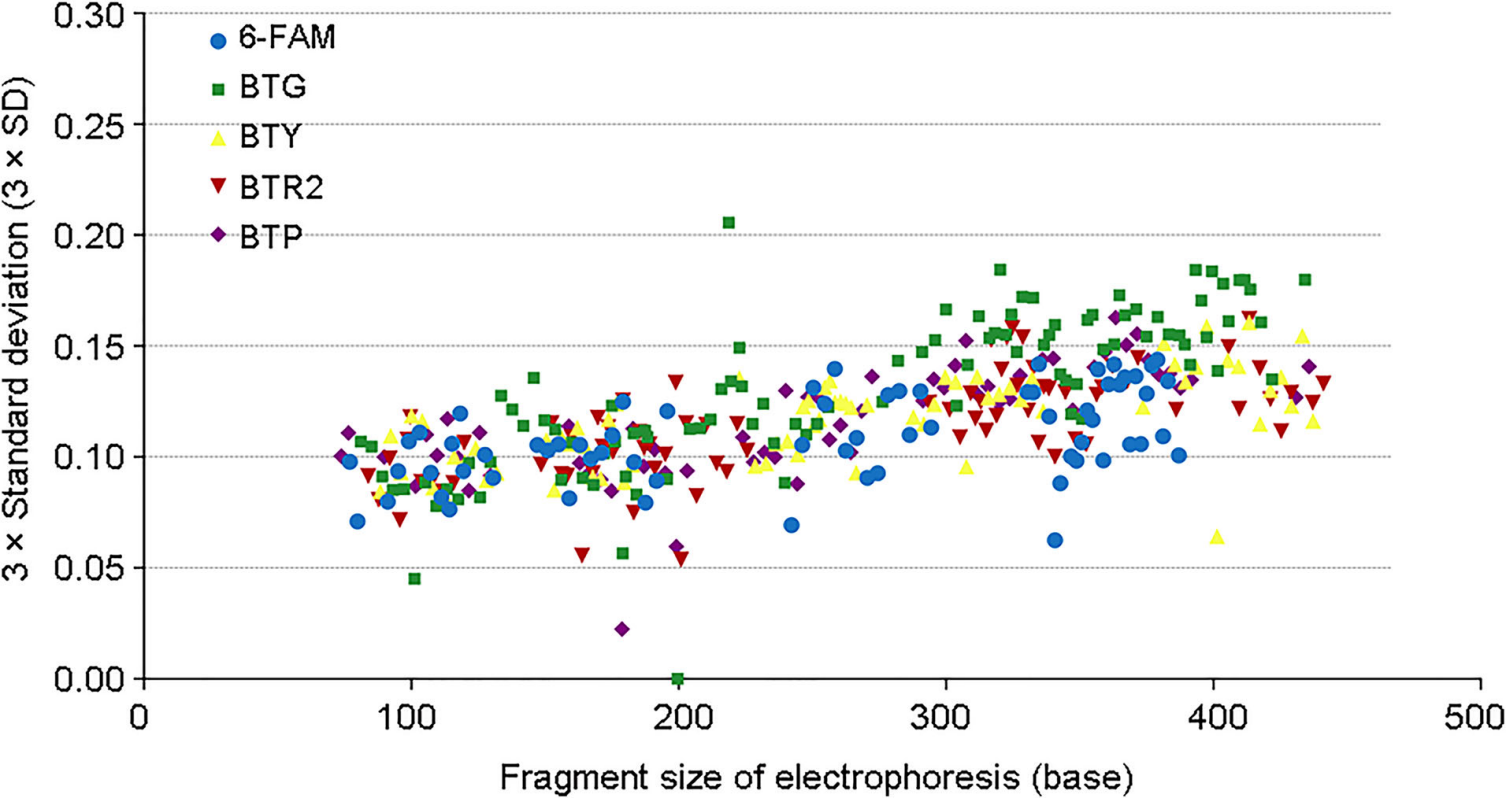
Precision study across 24 injections of the allelic ladder samples of the Investigator 24plex QS Kit. The average fragment sizes are plotted against 3 × standard deviation (3 × SD).

A sizing precision study is necessary for reliable and accurate genotyping. Based on the sizing data of the 24 allelic ladder samples, the average fragment sizes were plotted against the 3 × standard deviation (3 × SD), and the largest standard deviation was 0.0685 for D12S391 at allele 17.3 (Figure 1). In addition, the 24 allelic ladder samples were even equivalent (199.64 bp) for allele 20.3 at the D1S1656 locus.

**Figure 2.**
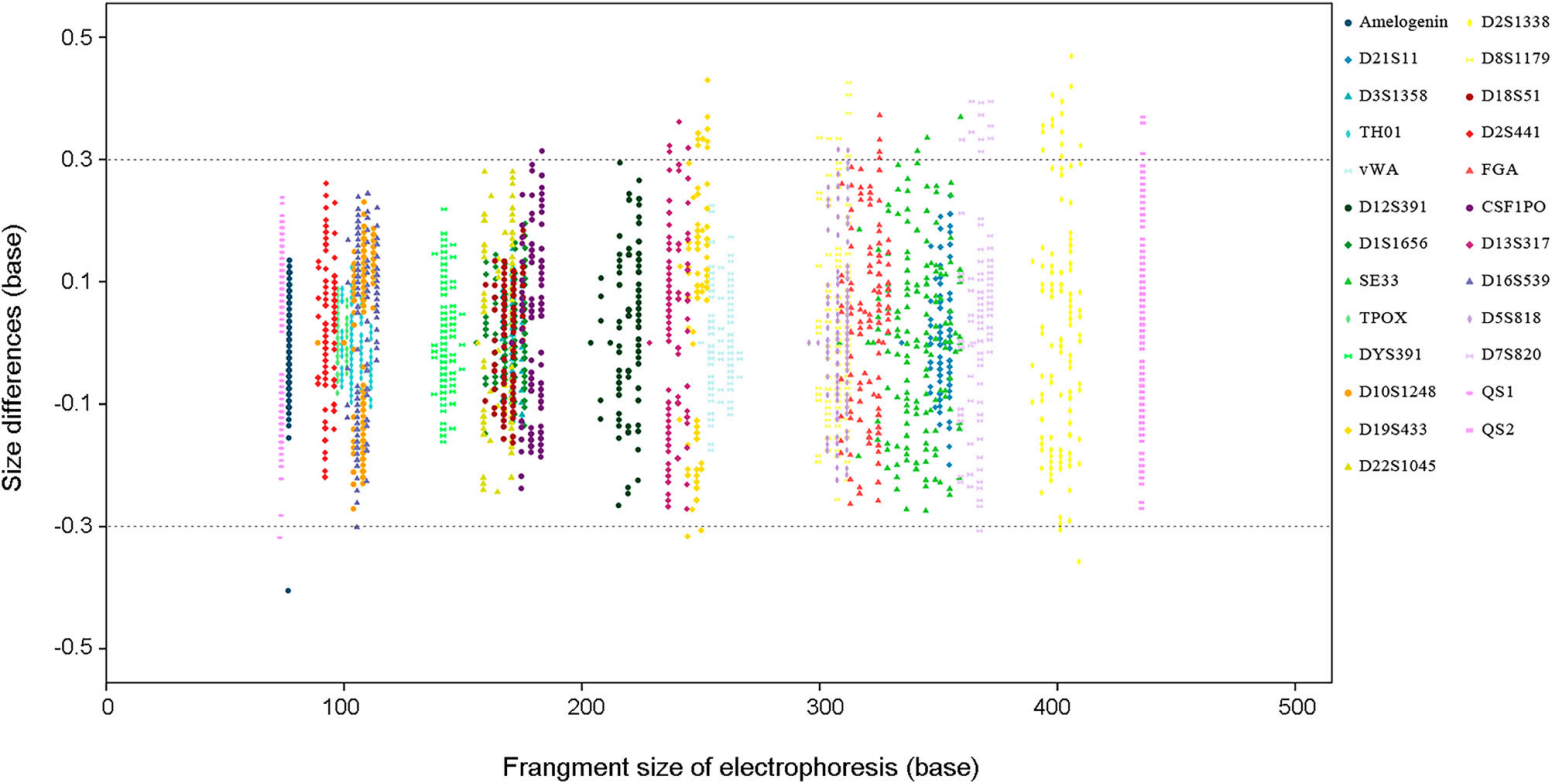
In the accuracy study, a total of 8 461 alleles from 200 random individual DNA samples are shown. The X-axis represents the fragment sizes and the Y-axis represents the size difference of each sample allele with the corresponding allelic ladder. All these alleles are presented within ±0.5 bases of a corresponding allelic ladder, mostly within a ±0.3 base window (the dotted lines parallel to X-axis).

The accuracy was evaluated by calculating the size differences of all 8 461 alleles from the 200 DNA samples compared to the corresponding allelic ladder. All the sample alleles were observed to be within ±0.5 bases of a corresponding allele, most of which were within ±0.3 bases (Figure 2). This result demonstrates that the multiplex system has sufficient sizing accuracy and that there is little risk of error in micro-variant allele genotyping.

### Repeatability/reproducibility, “forensic-type” samples and concordance testing

The genotyping profiles of the 50 individual DNA samples from the replicate tests of the 24plex QS Kit in our laboratory and from the tests reproduced in an independent accredited laboratory were consistent, which indicated the repeatability and reproducibility of this kit. Moreover, the genotypes of the 50 DNA samples from the 24plex system were fully concordant with those generated using the GlobalFiler™ kit, and no null alleles were obtained, which further illustrated the concordance and accuracy of the 24plex kit.

We further demonstrated that the 24plex kit could successfully genotype a batch of genuine “forensic-type” samples, including hair, nail, and those mentioned in *Section repeatability/reproducibility, “forensic-type” samples and concordance testing*. The genotype profile of the FFPEB sample is shown in Supplementary Figure S2, in which the allelic peak heights decrease with the increasing amplicon length, but the QS1 and QS2 peaks were not affected (the height ratio greater than 60%). The complete genotype profiles of a blood stain on an FTA card and a 10-year-old blood stain on gauze were also obtained through direct amplification by this kit. All these results illustrated that the Investigator 24plex QS Kit was robust and applicable in forensic casework.

### Sensitivity

**Figure 3.**
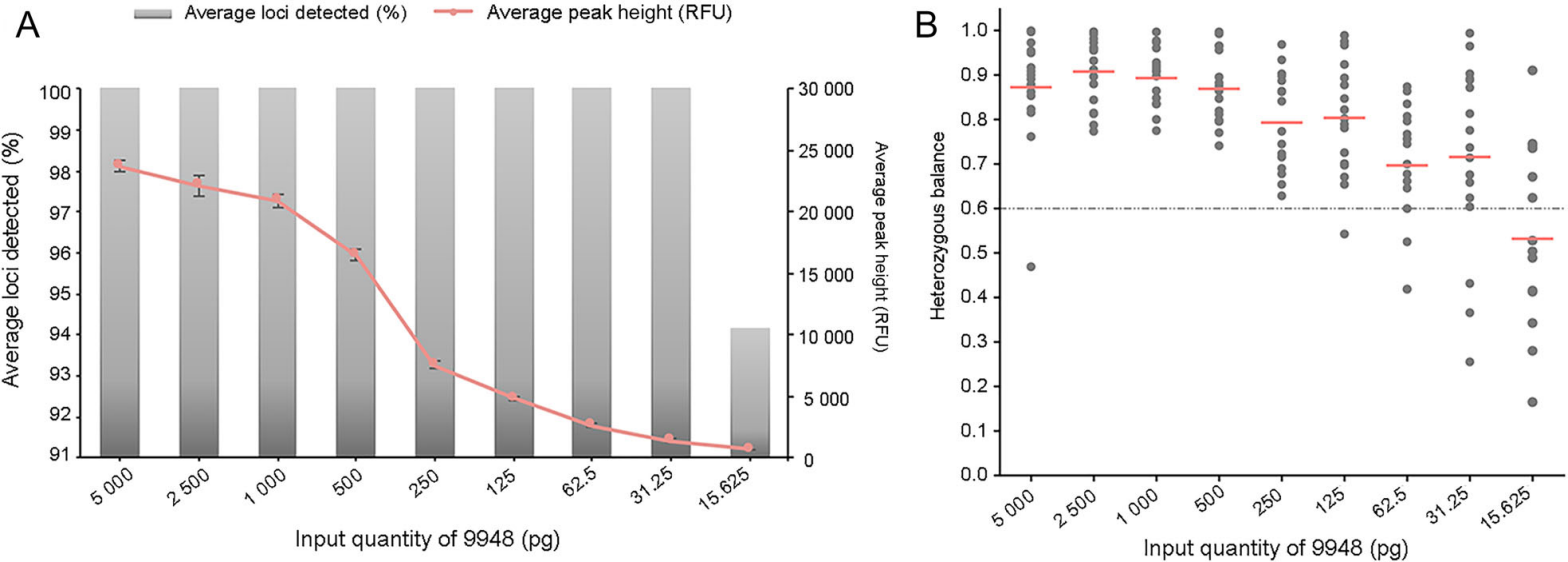
In the sensitivity study, control 9948 DNA ranging from 5 ng to 15.625 pg was amplified by the Investigator 24plex QS Kit. (A) Histogram and plots of the average percent of the detected loci (left Y-axis) and the average peak height (right Y-axis) of the 23 markers against DNA input quantity, respectively. Error bars represent the standard deviations in triplicate. (B) The heterozygous balance value of each heterozygous locus is plotted against serial dilutions of input DNA. The pink line is the average heterozygous balance value for each dilution and the horizontal dotted line indicates the reference line as 0.6.

A sensitivity study was performed to determine the range of the input DNA quantities that would generate reliable genotyping profiles and to confirm the detection limit of the present system. Triplicate results of serially diluted control DNA from the 9948 human cell line illustrated that the average peak heights proportionally decreased with the decreasing quantity of template DNA. Complete profiles could be recovered with as little as 31.25 pg DNA (Figure 3A). The average peak heights fluctuated from 23 749.98 RFU to 1 411.36 RFU as the input DNA ranged from 5 ng to 31.25 pg, and a further decrease of the quantity of template DNA (15.625 pg) produced a 5.83% allelic dropout with a lower 656.25 RFU average peak height. In previous studies [8], the Investigator 24plex QS Kit recovered 100% of alleles with at least 125 pg input DNA, while it was demonstrated to be more sensitive (31.25 pg) in our study, which may be the result of using the optimal electrophoresis and instrument status. Moreover, as shown in Figure 3B, the heterozygous balance values of the corresponding markers generally decreased with decreasing quantities of input DNA. When the input DNA ranged from 5 ng to 125 pg, all the heterozygous balance values were greater than 0.6, with only two exceptions at the D10S1248 locus at 5 ng and 125 pg. Spectral bleed-through was observed at 5 ng and 2.5 ng. Therefore, our results recommend a DNA input range between 1 ng and 125 pg DNA.

### Stability test

**Figure 4.**
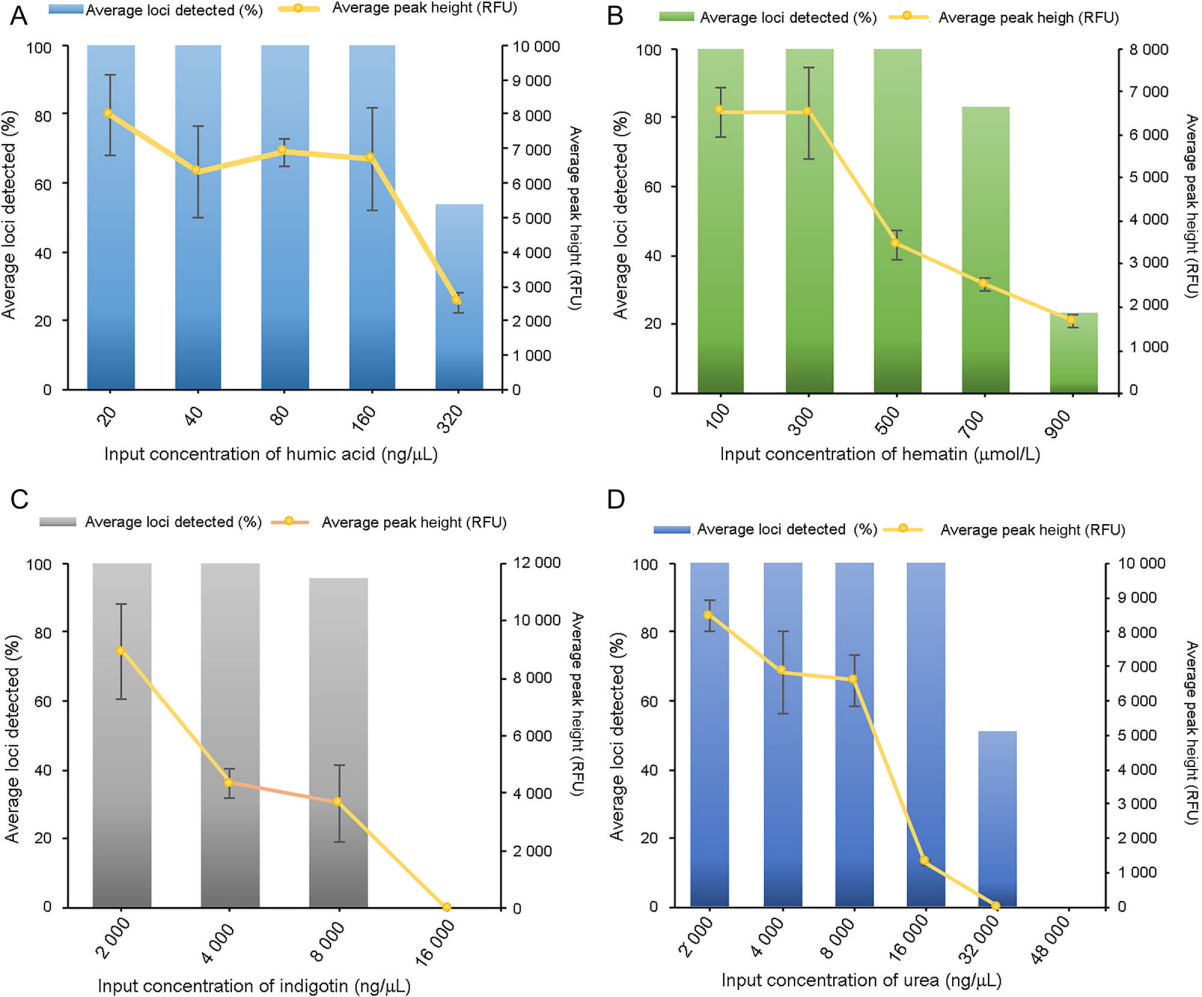
In the stability study, the average detected loci and average peak height were obtained by amplifying 1 ng of control 9948 DNA accompanied with increasing concentrations of (A) humic acid, (B) hematin, (C) indigotin and (D) urea using the Investigator 24plex QS Kit.

The ability of the Investigator 24plex QS Kit to obtain genotyping profiles from DNA subjected to common PCR inhibitors was assessed. As shown in Figure 4, for the hematin, humic acid, urea and indigotin groups, both the average percentage of loci detected and the average allele peak height decreased as the inhibitor concentrations increased. Full profiles were obtained at ≤500 µmol/L hematin, ≤160 ng/µL humic acid, ≤16 000 ng/µL urea, and ≤4 000 ng/µL indigotin, which is a significant improvement compared to many other CE-STR kits [14–16]. For nigrosine, concentrations of 40 ng/µL to 150 ng/µL produced profiles with a slightly altered peak height (9 070.43 – 10 387.76 RFU) but with several non-product peaks of fragments 85–120 bp in length. This was also observed in our previously established SiFaSTR™ 23-plex system (data not shown) and may be due to the influence of nigrosine on metal ions as well as some small molecules and enzymes involved in PCR [17]. In terms of the quality sensors, the peak height of QS2 was generally reduced more than that of QS1 in each concentration test of hematin, humic acid, urea, and indigotin. However, the peak height of the two quality sensors remained similar in the nigrosine group. At concentrations ≥320 ng/µL humic acid, ≥8 000 ng/µL indigotin, ≥32 000 ng/µL urea, and ≥700 µmol/L hematin, the QS2 peaks dropped out. The QS1 peaks, along with PCR progress, were completely inhibited at ≥16 000 ng/µL indigotin and ≥48 000 ng/µL urea.

### Species specificity

The DNA samples described in *Section species specificity* were amplified and assessed as an evaluation of the system’s potential cross-reactivity. The QS1 and QS2 loci were observed to be normal in profiles of all the non-human samples, which confirmed successful PCR amplification. Apart from these results, no recognizable peaks above 100 RFU were found except from the deer, snake and chicken groups.

As shown in Supplementary Figure S3(A), two “OL” peaks (peak height 1 012 and 1 069 RFU, respectively) at the D5S818 locus in BTP dye with sizes of 329.54 bp and 331.14 bp were detected in chicken DNA. Additionally, there were two “OL” peaks (peak height 224 and 223 RFU, respectively) observed at the D10S1248 locus in BTY dye with sizes of 84.12 bp and 91.66 bp, respectively in deer DNA (Supplementary Figure S3(B)). Snake DNA generated a 92.49 bp “OL” peak (peak height 419 RFU) at the D16S539 locus in BTP dye (Supplementary Figure S3(C)). Although several peaks located outside of each bin set were detected, the genotyping results were not affected. Overall, the species specificity results indicated that the Investigator 24plex QS Kit is human-specific.

### Mixture study

Forensic evidence samples that contain body fluids or secretions from two or more individuals are common in forensic cases [18]. Thus, it is vital to evaluate the ability of STR typing kits to detect a DNA mixture, especially for components from minor contributor. In our study, male and female DNA (9947A and 9948 human cell lines, respectively) or DNA from two individual males (9948 and 2800M cell lines) was mixed to yield 1 ng DNA input at various ratios, which were amplified in triplicate using the 24plex kit. The genotype profiles of 9947A/9948 and 9948/2800M at a mixed ratio of 1:1 are shown in Supplementary Figure S4. As the minor component DNA reduced, a corresponding drop in peak height occurred for its alleles. As a whole, full profiles (59 alleles for 9947A/9948 and 66 alleles for 9948/2800M) were obtained at mixed ratios ranging from 1:1 to 1:19 (19:1) for each triplicate test, which was consistent with the sensitivity study. The QS1/QS2 peaks remained unaffected. However, when the mixed ratio reached 1:19 or 19:1, the alleles of the minor contributor was indistinguishable from the stutter peak of the major contributor’s alleles, and in this situation, the reference genotype profiles are helpful.

### Balance and stutter determination

For accurate heterozygote genotyping and low-template or degraded sample detection, the intra-locus, intra-colour, and inter-colour balances are recommended to be ≥0.7, ≥0.5, and ≥0.3, respectively [10,19]. As shown in Table 1, based on the allele height data of 200 DNA samples, the intra-locus balance values of the 22 loci in the kit (except for the homozygous DYS391) ranged from 0.8366 (vWA) to 0.8849 (TPOX). The intra-colour balance values of the five different florescent dyes ranged from 0.8592 (BTG) to 0.9457 (BTR2), and the value of inter-colour balance was 0.8967. These values fully satisfy the aforementioned established standards and even exceed many other STR typing systems [11,15,20].

**Table 1.** Balance calculation for the Investigator 24plex QS Kit.

	Counts	Mean	SD	Min	Max
**Intra-locus**					
Amelogenin	100	0.8816	0.1075	0.4199	0.9999
TH01	134	0.8709	0.1053	0.2422	0.9992
D3S1358	136	0.8733	0.0886	0.5596	0.9996
vWA	155	0.8366	0.1149	0.3895	0.9989
D21S11	170	0.8667	0.1008	0.5304	0.9972
TPOX	130	0.8849	0.1034	0.3674	0.9964
D1S1656	158	0.8681	0.0976	0.4497	0.9985
D12S391	170	0.8634	0.1045	0.4015	1.0000
SE33	185	0.8520	0.1156	0.4992	0.9994
D10S1248	147	0.8624	0.0932	0.5156	0.9983
D22S1045	161	0.8543	0.1027	0.5247	0.9986
D19S433	157	0.8801	0.0826	0.6454	0.9992
D8S1179	175	0.8838	0.0866	0.5786	0.9994
D2S1338	174	0.8587	0.1016	0.4583	0.9988
D2S441	146	0.8815	0.0863	0.6274	0.9995
D18S51	167	0.8786	0.0864	0.5583	0.9994
FGA	169	0.8561	0.0985	0.3899	0.9989
D16S539	159	0.8819	0.0935	0.5149	0.9988
CSF1PO	152	0.8756	0.1028	0.4750	0.9994
D13S317	152	0.8803	0.0974	0.4921	0.9991
D5S818	151	0.8846	0.0823	0.5588	0.9985
D7S820	158	0.8617	0.1223	0.3603	0.9974
**Intra-colour**					
FAM		0.9385	0.0545	0.8724	0.9958
BTG		0.8592	0.1618	0.6171	0.9534
BTY		0.9221	0.0524	0.8392	0.9759
BTR2		0.9457	0.0321	0.9106	0.9736
BTP		0.9110	0.0978	0.7929	0.9914
**Inter-colour**		0.8967	0.0898	0.4161	0.9993

The balance parameters of intra-locus, intra-colour and inter-colour were calculated based on the genotyping results of 200 individuals (male = 100, female = 100). The balance criteria were as follows: intra-locus balance above 0.7, intra-colour balance above 0.5 and inter-colour balance above 0.3. SD: standard deviation.

Stutter peaks are considered to be the additional PCR products that result from strand slippage [21]. A stutter peak usually manifests as one repeat unit smaller or larger than the associated main allele peak (*n* ± 3 for trinucleotide and *n* ± 4 for tetranucleotide) [3,22]. In terms of the stutter analysis for the 24plex system, only minus stutter peaks were taken into consideration as few plus stutters were detected among these samples. The peak heights of the alleles and the corresponding stutters of these 200 DNA samples were used to calculate the stutter percentage and SD. As shown in Table 2, the tetranucleotide-repeat locus SE33 represented the highest average stutter ratio (12.43%), and the lowest was observed at the tetranucleotide-repeat locus TH01 (3.82%); these results agree with reports from Singapore and the US population sets [8,23]. The stutter filter was defined by the average percentage of stutter plus or minus 3×SD, which is also considered to be significant in mixture interpretation.

**Table 2.** Stutter analysis of the 23 loci in the Investigator 24plex QS Kit.

Locus	Minus stutter		
	Stutter percentage	SD	Stutter filter^a^
TH01	0.0382	0.0143	0.0811
D3S1358	0.1106	0.0207	0.1726
vWA	0.0911	0.0256	0.1679
D21S11	0.0973	0.0193	0.1553
TPOX	0.0508	0.0307	0.1429
DYS391	0.0750	0.0140	0.1171
D1S1656	0.1183	0.0301	0.2085
D12S391	0.1229	0.0312	0.2165
SE33	0.1243	0.0279	0.2080
D10S1248	0.1042	0.0183	0.1590
D22S1045	0.1202	0.0464	0.2594
D19S433	0.0847	0.0158	0.1321
D8S1179	0.0895	0.0187	0.1457
D2S1338	0.1049	0.0198	0.1643
D2S441	0.0702	0.0264	0.1494
D18S51	0.0971	0.0257	0.1741
FGA	0.0863	0.0202	0.1469
D16S539	0.0829	0.0253	0.1588
CSF1PO	0.0855	0.0330	0.1846
D13S317	0.0619	0.0270	0.1430
D5S818	0.0771	0.0239	0.1487
D7S820	0.0610	0.0152	0.1066

Stutter values were obtained from genotyping data of 200 individual samples. The analytical threshold of the minimum stutter peak height was set to 20 RFUs.

SD: standard deviation.

^a^ Stutter filter = mean ± 3 × SD (shown as decimal value).

### Population study

In the present study, a total of 500 unrelated Chinese Han individuals (250 males and 250 females) were successfully genotyped using the 24plex system. A total of 279 alleles at 22 STR loci were observed with allelic frequencies spanning from 0.001 to 0.500. According to the exact Chi square test, there was a statistically significant deviation from HWE at SE33 (*P* < 0.05) in the studied population, which could be the result of a bias of the sample set. Although, after applying Bonferroni's correction, no significant deviation was observed, adding more unrelated individuals might be necessary in the further study. No significant LD was observed between these STR loci in the studied population after Bonferroni’s correction. The HET values ranged from 0.6280 (TPOX) to 0.9180 (SE33); correspondingly, the PIC of these two loci were the lowest (0.5614) and highest (0.9413), respectively. In total, these 21 autosomal STR loci showed a high forensic efficiency with average PIC values of no less than 0.7629 and TDP, CPE_D,_ and CPE_T_ values exceeding 0.999 999 999 9, 0.999 997 225 8 and 0.999 999 999 5, respectively. The GD value of the DYS391 locus among the 250 Han males was 0.4291, while the HMP and DC were 0.5726 and 0.0240, respectively. The allelic frequencies and forensic parameters of these 22 STR loci are listed in Supplementary Table S2, which illustrates that the STR loci contained in the Investigator 24plex QS Kit were highly informative and useful for human identification and paternity testing in Chinese Han individuals.

## Conclusion

In our study, we conducted a pilot validation study of the Investigator 24plex QS Kit in the Chinese Han population. One of the special features of this kit is the inclusion of two innovative internal performance controls, QS1 and QS2, which evaluate the PCR performance and can aid in troubleshooting. This 6-dye DNA profiling system was found to be highly sensitive and reliable for genotyping various forensic-type samples and could tolerate common PCR inhibitors. The population investigation demonstrated its applicability in forensic genetics among the Chinese Han population, which also provided useful population STR data that enriched the STR database.
